# Supplementary material for: Parental perceptions of children’s physical activity participation: An exploration of satisfaction, school-based engagement, barriers to participation and preferred strategies for improvement
Source: PLoS One. 2026 Sep 16;21(9):e0351523. doi: 10.1371/journal.pone.0351523 (PMC13581000; doi:10.1371/journal.pone.0351523)
Supplement: Supplementary file 1 [file pone.0351523.s002.docx]

**RESPONSE TO THE ACADEMIC EDITOR AND REVIEWERS**

**Manuscript ID:** PONE-D-26-26322

**Title:** *Parental perceptions of children's physical activity participation: an exploration of satisfaction, school-based engagement, barriers to participation and preferred strategies for improvement*

Dear Dr. Morufu Olalekan Raimi,

We sincerely thank you and the reviewers for your careful evaluation of our manuscript and for the constructive comments and suggestions. We appreciate the time and effort invested in reviewing our work. We have carefully considered all comments and have revised the manuscript accordingly. We believe that these revisions have substantially strengthened the methodological transparency, reporting quality, and overall clarity of the manuscript.

A detailed point-by-point response to each comment is provided below. Changes made in the manuscript are highlighted in the tracked-changes version submitted alongside the revised manuscript.

**Response to Editor**

***Comment 1***

*Resolve internal inconsistencies in author contributions and the data collection team.*

**Response**

We thank the Academic Editor for identifying these inconsistencies. The manuscript has been revised to ensure complete consistency between the Data Collection Procedures section and the Authors' Contributions statement. The composition of the data collection team has been standardised throughout the manuscript, author initials have been corrected and standardised, and the Authors' Contributions section has been updated to accurately reflect the specific roles performed by each author.

**Changes made**

- Revised the Data Collection Procedures section.
- Revised the Authors' Contributions section.
- Standardised author initials throughout the manuscript.

***Comment 2***

*Clarify the role of Juliet Lucy Ekowa.*

**Response**

Thank you for this important observation. The Authors' Contributions section has been revised to explicitly describe Juliet Lucy Ekowa's contributions. Her contributions now include participation in the development and refinement of the research questions and study design, as well as data analysis and interpretation, thereby ensuring compliance with the PLOS ONE authorship criteria.

**Changes made**

- Revised the Authors' Contributions section.

***Comment 3***

*Provide a clearer justification of sample size and thematic sufficiency.*

**Response**

We appreciate this valuable suggestion. The Participant Selection and Sampling Strategy section has been expanded to describe how thematic sufficiency was operationalised during data collection. We now explain that interview transcripts were reviewed alongside ongoing data collection, preliminary coding and discussion of emerging concepts were undertaken iteratively by the research team, and recruitment ceased when successive interviews yielded no substantially new themes, with later interviews contributing additional depth rather than novel conceptual insights.

**Changes made**

- Expanded the Participant Selection and Sampling Strategy section.

***Comment 4***

*Address mixed interview modality as a methodological consideration.*

**Response**

We thank the Academic Editor for this helpful recommendation. The Methods section has been revised to clarify the consent procedures for participants interviewed by telephone. Specifically, participant information sheets and consent forms were sent electronically via WhatsApp before the scheduled interview, and signed consent forms were returned before interviews commenced. We have also expanded the Strengths and Limitations section to acknowledge the potential influence of mixed interview modalities on data richness and the inability to observe non-verbal cues during telephone interviews.

**Changes made**

- Revised the Data Collection Procedures section.
- Revised the Strengths and Limitations section.

***Comment 5***

*Provide practical details of the analytic process.*

**Response**

We appreciate this constructive suggestion. The Data Analysis section has been substantially expanded to provide greater methodological transparency. We now describe the use of manual coding, iterative coding procedures, collaborative reflexive discussions among members of the research team, theme development and refinement, and clarify that qualitative data were analysed using Braun and Clarke's Reflexive Thematic Analysis. We also clarify that qualitative data analysis software was not used and that member checking was not undertaken, consistent with the adopted methodological approach.

**Changes made**

- Revised the Data Analysis section.

***Comment 6***

*Strengthen discussion of sample skew and transferability.*

**Response**

Thank you for this insightful recommendation. We have strengthened the Discussion by explicitly acknowledging that the study sample consisted predominantly of highly educated parents employed in the government sector, many of whom had children attending private schools. We now discuss how these characteristics may influence the transferability of the findings and caution that the identified barriers and facilitators may differ among lower socioeconomic, rural, or public-school populations. Similar clarification has also been incorporated into the Strengths and Limitations section.

**Changes made**

- Revised the Discussion.
- Revised the Strengths and Limitations section.

***Comment 7***

*Address minor reporting issues.*

**Response**

We appreciate these helpful suggestions. In response, we removed duplicated reflexivity statements, deleted unused abbreviations, revised repetitive theme-closing summary statements to improve readability, strengthened reflexivity reporting in accordance with the COREQ guidance, and confirmed that a completed COREQ checklist has been included as Supporting Information. The manuscript has also undergone careful editing to improve clarity, consistency, and overall reporting quality.

**Changes made**

- Revised multiple sections throughout the manuscript.
- Updated the abbreviations list.
- Included the completed COREQ checklist as Supporting Information.

**Comment 8**

*Please comply with the PLOS data availability policy. The online submission indicates that de-identified qualitative data are available from the corresponding author upon reasonable request. However, PLOS requires all data underlying the findings to be made freely available, either in a public repository, within the manuscript, or as Supporting Information.*

**Response**

Thank you for this important comment. We have revised the submission to comply with the PLOS data availability policy. The de-identified qualitative data underlying the findings have now been uploaded as S2 File (Supporting Information). All direct and indirect identifying information has been removed to protect participant confidentiality while ensuring transparency and reproducibility of the study. The Data Availability Statement has also been revised to indicate that the de-identified qualitative data are available as Supporting Information.

**Changes made**

- Uploaded the de-identified qualitative dataset as S2 File (Supporting Information).
- Revised the Data Availability Statement to reflect that the qualitative data are publicly available as Supporting Information.

**Response to Reviewer 1**

We sincerely thank the reviewer for the positive evaluation of our manuscript and for the constructive comments and suggestions. We appreciate the recognition of the importance of our study and have carefully considered each recommendation. The manuscript has been revised accordingly, as detailed below.

***Comment 1***

*The paper is clear and well-structured, with an important topic about parental perceptions of children's physical activity.*

**Response**

We sincerely thank the reviewer for the positive assessment of our work and for recognising the importance and relevance of the study. We appreciate the encouraging comments and have carefully addressed all subsequent recommendations to further strengthen the manuscript.

**Changes made**

- We carefully revised the manuscript in response to all reviewer and editor comments to improve methodological transparency, reporting quality, and overall clarity.

**Comment 2**

*Small inconsistency in author initials for data collection – please check and correct.*

**Response**

Thank you for identifying this inconsistency. We carefully reviewed the manuscript and standardised the author initials throughout. We also reconciled the descriptions of the data collection team and revised the Authors' Contributions section to ensure consistency across all sections of the manuscript.

**Changes made**

- Standardised author initials throughout the manuscript.
- Revised the Data Collection Procedures section.
- Revised the Authors' Contributions section.

**Comment 3**

*Timeline of interviews (2025 vs. submission in 2026) needs a short clarification.*

**Response**

We appreciate this helpful suggestion. The manuscript has been revised to clarify the study timeline. We now explicitly state that interviews were conducted between August and October 2025, followed by transcription, data analysis, and interpretation between November 2025 and early 2026 prior to manuscript preparation and submission.

**Changes made**

- Revised the Data Collection Procedures section to clarify the study timeline.

**Comment 4**

*The sample is mainly educated parents with children in private schools – it would help to note that strategies may need adaptation for public schools.*

**Response**

Thank you for this valuable observation. We agree that the socioeconomic characteristics of the study sample may influence the applicability of the findings to other settings. Accordingly, we have strengthened the Discussion by explicitly acknowledging that the identified barriers, facilitators, and recommended strategies may differ among families from lower socioeconomic backgrounds, rural communities, and those with children attending public schools. We have also incorporated this consideration into the Strengths and Limitations section to provide a more balanced interpretation of the findings.

**Changes made**

- Revised the Discussion section.
- Revised the Strengths and Limitations section.

**Comment 5**

*Adding one sentence on how clinicians (e.g., pediatric physiotherapists) can use these insights in practice would strengthen the conclusion.*

**Response**

We appreciate this excellent suggestion. In response, we have expanded the "Implications for Practice and Policy" section to include the clinical relevance of the findings for paediatric physiotherapists and other child health professionals. We have also revised the Conclusion to highlight how these findings can inform the design and implementation of family-, school-, and community-based interventions aimed at promoting children's physical activity.

**Changes made**

- Added clinical implications to the "Implications for Practice and Policy" section.
- Revised the Conclusion to highlight the implications for paediatric physiotherapists, educators, public health practitioners, and other child health professionals.

**Response to Reviewer 2**

We sincerely thank the reviewer for the thorough and constructive evaluation of our manuscript. We appreciate the positive comments regarding the importance of the research topic, the appropriateness of the qualitative design, and the coherence of the findings. We have carefully considered each comment and have revised the manuscript accordingly. Our detailed responses are provided below.

**General Comment**

*The study addresses an under-researched question and applies an appropriate qualitative design and an explicit theoretical framework (the Socio-Ecological Model). The five themes are coherent and well illustrated with participant quotations.*

**Response**

We sincerely thank the reviewer for these encouraging comments and for recognising the importance of the study, the suitability of the qualitative design, and the application of the Socio-Ecological Model. We appreciate the constructive recommendations provided and have revised the manuscript to improve methodological transparency, reporting quality, and interpretation of the findings.

**Major Comments**

**Comment 1**

*Inconsistent reporting of the data-collection team.*

**Response**

We thank the reviewer for identifying this important inconsistency. The manuscript has been carefully reviewed and revised to ensure that the composition of the data collection team is reported consistently throughout. The Data Collection Procedures, Researcher Positionality, and Authors' Contributions sections have all been updated to accurately reflect the roles of the researchers involved in the qualitative study.

**Changes made**

- Revised the Data Collection Procedures.
- Revised the Researcher Positionality section.
- Revised the Authors' Contributions section.
- Standardised author initials throughout the manuscript.

**Comment 2**

*Inconsistent author initials for one co-author.*

**Response**

Thank you for bringing this to our attention. We have standardised the initials of Adaeze I. Onyekwelu throughout the manuscript to ensure consistency across all sections, including the Authors' Contributions statement.

**Changes made**

- Standardised author initials throughout the manuscript.

**Comment 3**

*Incomplete contribution statement for one listed author.*

**Response**

We appreciate this important observation. The Authors' Contributions section has been revised to clearly specify Juliet Lucy Ekowa's contributions to the study. Her role now includes contributing to the development and refinement of the research questions and study design, as well as participation in data analysis and interpretation, thereby ensuring compliance with the PLOS ONE authorship criteria.

**Changes made**

- Revised the Authors' Contributions section.

**Comment 4**

*Justification of sample size and thematic sufficiency.*

**Response**

We thank the reviewer for this valuable recommendation. The Participant Selection and Sampling Strategy section has been expanded to provide a clearer description of how thematic sufficiency was determined during data collection. Specifically, we now explain that interview transcripts were reviewed alongside ongoing interviews, preliminary coding and discussion of emerging concepts were undertaken iteratively by the research team, and recruitment ceased when successive interviews yielded no substantially new themes, with later interviews contributing additional depth and clarification rather than novel conceptual insights.

**Changes made**

- Expanded the Participant Selection and Sampling Strategy section.

**Comment 5**

*Mixed interview modality not addressed as a methodological consideration.*

**Response**

We appreciate this helpful suggestion. The Methods section has been revised to clarify the procedures used for telephone interviews, including the electronic distribution and return of participant information sheets and signed consent forms before interviews commenced. We have also expanded the Strengths and Limitations section to acknowledge that the use of both telephone and face-to-face interviews may have influenced data richness because telephone interviews limited the observation of non-verbal cues, while also improving accessibility and participation among parents with demanding schedules.

**Changes made**

- Revised the Data Collection Procedures.
- Revised the Strengths and Limitations section.

**Comment 6**

*Analytic procedure needs more detail.*

**Response**

We thank the reviewer for this constructive recommendation. The Data Analysis section has been substantially expanded to provide greater methodological transparency. We now specify that coding was undertaken manually, describe the iterative coding process, explain the collaborative reflexive discussions undertaken by the research team during code and theme development, and clarify that the analysis followed Braun and Clarke's Reflexive Thematic Analysis. We also clarify that qualitative data analysis software was not used and that participant (member) checking was not undertaken, consistent with the principles of reflexive thematic analysis.

**Changes made**

- Revised the Data Analysis section.

**Comment 7**

*Sample skew and generalisability.*

**Response**

We appreciate this insightful recommendation. The Discussion has been strengthened by explicitly acknowledging that the participant sample consisted predominantly of highly educated parents employed in the government sector, many of whom had children attending private schools. We now discuss how these characteristics may influence parental perceptions of children's physical activity and recognise that the findings may not be directly transferable to lower socioeconomic populations, rural communities, or families with children attending public schools. Similar clarification has also been incorporated into the Strengths and Limitations section.

**Changes made**

- Revised the Discussion.
- Revised the Strengths and Limitations section.

**Minor Comments**

**Comment 1**

*The title is rather long.*

**Response**

We appreciate the reviewer's suggestion. We carefully considered shortening the title but elected to retain it because we believe it accurately reflects the scope, objectives, and principal findings of the study while remaining informative for readers.

**Comment 2**

*Specify the recruitment settings in the abstract.*

**Response**

Thank you for this helpful suggestion. The Abstract has been revised to specify that participants were recruited purposively from community settings, including churches, marketplaces, shopping centres, and recreational areas within Enugu City.

**Changes made**

- Revised the Abstract.

**Comment 3**

*Theme-closing summaries are repetitive.*

**Response**

We appreciate this observation. The concluding summary statements for Themes 1, 3, and 4 have been revised to improve readability, reduce repetition, and strengthen the interpretive nature of the findings.

**Changes made**

- Revised the Results section.

**Comment 4**

*Discussion restates the Results.*

**Response**

Thank you for this constructive recommendation. The Discussion has been revised to reduce repetition of the Results section and place greater emphasis on interpretation, integration with previous literature, and implications for practice and policy.

**Changes made**

- Revised the Discussion.

**Comment 5**

*Unused abbreviations.*

**Response**

Thank you for identifying this issue. We reviewed the abbreviations list and removed abbreviations that were not used in the manuscript to improve clarity and consistency.

**Changes made**

- Revised the abbreviations list.

**Comment 6**

*COREQ checklist.*

**Response**

Thank you for this reminder. A completed and updated COREQ checklist has been prepared and included as Supporting Information. The checklist has been reviewed to ensure consistency with the revised manuscript.

**Changes made**

- Submitted the completed COREQ checklist as Supporting Information.

**Comment 7**

*Duplicate reflexivity statement.*

**Response**

We appreciate the reviewer's careful reading of the manuscript. The duplicated statement regarding reflexive discussions and peer debriefing has been removed, and the relevant sections have been revised to improve clarity while avoiding unnecessary repetition.

**Changes made**

- Revised the Researcher Positionality and Data Analysis sections.

**General Response**

We sincerely thank the Academic Editor and both reviewers for their thoughtful and constructive comments. Their suggestions have substantially improved the methodological transparency, reporting quality, and overall clarity of our manuscript. We believe that the revised manuscript addresses all concerns raised and hope that it is now suitable for publication in PLOS ONE.
