# Supplementary material for: Parental perceptions of children’s physical activity participation: An exploration of satisfaction, school-based engagement, barriers to participation and preferred strategies for improvement
Source: PLoS One. 2026 Sep 16;21(9):e0351523. doi: 10.1371/journal.pone.0351523 (PMC13581000; doi:10.1371/journal.pone.0351523)
Supplement: Supplementary file 2 [file pone.0351523.s003.docx]

**RESPONSE TO THE ACADEMIC EDITOR**

**Manuscript ID:** PONE-D-26-26322

**Title:** *Parental perceptions of children's physical activity participation: an exploration of satisfaction, school-based engagement, barriers to participation and preferred strategies for improvement*

Dear Dr. Morufu Olalekan Raimi,

We sincerely thank the Academic Editor for the careful evaluation of our manuscript and for the constructive comments. We greatly appreciate the positive assessment of our work and the opportunity to further improve the manuscript. We have carefully considered all comments. Comments that had already been fully addressed in our previous revision (Revision 1) have been retained in the manuscript and therefore do not appear as new tracked changes in the current revision. The remaining comments have been addressed in the current revision. Our detailed responses are provided below.

**Revision A: Resolve Remaining Author Contribution Inconsistencies (Critical)**

**Comment**

The manuscript contained inconsistencies regarding which authors conducted the data collection. Please reconcile these discrepancies and clearly specify the contributions of all authors, particularly Juliet Lucy Ekowa.

***Response***

*Thank you for identifying this important issue. The major inconsistencies regarding author contributions were addressed in our previous revision (Revision 1). In response to the current comment, we further reviewed the manuscript to ensure complete consistency across all relevant sections. The Data Collection Procedures section now consistently states that data collection was conducted by* ***CO, OJ, CE, and AO****, with CO serving as the primary interviewer and OJ, CE, and AO supporting the interviews through field notes, interview coordination, and follow-up probing where appropriate. The Data Analysis section now clearly specifies that* ***KF and CO*** *undertook the initial manual coding, while* ***CO, KF, AO, and JE*** *participated in reflexive discussions, interpretation of findings, refinement of codes, and development of themes. The Authors' Contributions section has also been aligned with these descriptions and now explicitly describes Juliet Ekowa's contribution to study design, thematic analysis, interpretation of findings, and refinement of themes.* **(Data Collection Procedures: line 187-192. page 8, Data Analysis: line 230-232. Page 10, Authors' Contributions: line 540-544. Page 26)**

**Revision B: Remove Duplicate Text in the Data Analysis Section**

**Comment**

The Data Analysis section contained duplicated descriptions of the Braun and Clarke six-phase framework.

***Response***

*Thank you for this observation. This issue was fully addressed in our previous revision (Revision 1). The duplicated description of the Braun and Clarke six-phase framework had already been removed, and the Data Analysis section now contains a single, coherent description of the analytical process. Consequently, no additional revisions were required in the current version.* **(Data Analysis: line 225-241. Page 10)**

**Revision C: Clarify Thematic Sufficiency**

**Comment**

Please clarify how thematic sufficiency was operationalised during data collection, including whether coding occurred alongside data collection, whether team consensus was used, and how the decision to stop recruitment was reached.

***Response***

*Thank you for this helpful suggestion. The Participant Selection and Sampling Strategy section has been further revised to provide a clearer description of how thematic sufficiency was operationalised. The manuscript now explains that interview transcripts were reviewed alongside ongoing interviews, preliminary coding and discussion of emerging concepts occurred concurrently with data collection, and the research team met regularly to review emerging codes. It further states that the research team reached consensus that thematic sufficiency had been achieved when successive interviews yielded no substantially new themes, with subsequent interviews providing additional depth and clarification rather than novel conceptual insights, consistent with the principles of reflexive thematic analysis.* **(Participant Selection and Sampling Strategy: line 163-171. page 7)**

**Revision D: Clarify Mixed Interview Modality and Telephone Consent**

**Comment**

Please clarify whether signed consent forms were returned before the interview and whether verbal consent was obtained at the beginning of telephone interviews.

***Response***

*Thank you for this valuable suggestion. The Data Collection Procedures section has been revised to provide a complete description of the consent procedures for telephone interviews. The manuscript now specifies that the participant information sheet and consent form were sent via WhatsApp before the scheduled interview, signed consent forms were returned electronically prior to the commencement of the interview, and verbal confirmation of informed consent was obtained at the beginning of each telephone interview before audio recording commenced.* **(Data Collection Procedures: line 192-201. page 9)**

**Revision E1: Parent Occupation Variable**

**Comment**

The occupation variable appeared in Table 1 but was not described in the Results.

***Response***

*Thank you for highlighting this omission. This comment was fully addressed in our previous revision (Revision 1). The Results section was revised to include a description of participants' occupational characteristics, indicating that most participants were employed within the government public sector (75%), while the remainder were self-employed or business owners. Therefore, no additional changes were required in the current revision.* **(Participant Characteristics: line 255-256. page 11)**

**Revision E2: Remove Unused Abbreviation**

**Comment**

Please remove the unused abbreviation "PHE".

***Response***

*Thank you for this observation. This issue was fully addressed in our previous revision (Revision 1). The unused abbreviation* ***PHE*** *was removed from the abbreviations list. Consequently, no further revision was required in the current manuscript.* **(Abbreviations list: line 533-535. page 26)**

**Revision E3: Remove Duplicate Reflexivity Statement**

**Comment**

Please remove the duplicated reflexivity statement.

***Response***

*Thank you for identifying this issue. This comment was fully addressed in our previous revision (Revision 1). The duplicated reflexivity statement had already been removed, and reflexivity is now described only within the appropriate methodological sections. Therefore, no additional revisions were required in the current version.* **(Researcher Positionality and Reflexivity: line 203-223. page 10)**

We sincerely appreciate the Academic Editor's thoughtful and constructive comments, which have helped us improve the methodological transparency, clarity, and overall quality of the manuscript. We hope that the revisions satisfactorily address all concerns and respectfully submit the revised manuscript for your further consideration.

Thank you once again for your time and valuable feedback. We look forward to your decision.
