# Supplementary material for: Parental perceptions of children’s physical activity participation: An exploration of satisfaction, school-based engagement, barriers to participation and preferred strategies for improvement
Source: PLoS One. 2026 Sep 16;21(9):e0351523. doi: 10.1371/journal.pone.0351523 (PMC13581000; doi:10.1371/journal.pone.0351523)
Supplement: Peer review history

# Peer review: Decision Letter 0

**DOI:** 10.1371/journal.pone.0351523.r001

**Authors:** Morufu Raimi

21 Jul 2026

Dear Dr. Fatai,

Thank you for submitting your manuscript to PLOS ONE. After careful consideration, we feel that it has merit but does not fully meet PLOS ONE’s publication criteria as it currently stands. Therefore, we invite you to submit a revised version of the manuscript that addresses the points raised during the review process.

Please submit your revised manuscript by Sep 19 2026 11:59PM. If you will need more time than this to complete your revisions, please reply to this message or contact the journal office at plosone@plos.org. When you're ready to submit your revision, log on to https://www.editorialmanager.com/pone/ and select the 'Submissions Needing Revision' folder to locate your manuscript file.

- A letter that responds to each point raised by the academic editor and reviewer(s). You should upload this letter as a separate file labeled 'Response to Reviewers'.
- A marked-up copy of your manuscript that highlights changes made to the original version. You should upload this as a separate file labeled 'Revised Manuscript with Track Changes'.
- An unmarked version of your revised paper without tracked changes. You should upload this as a separate file labeled 'Manuscript'.

If you would like to make changes to your financial disclosure, please include your updated statement in your cover letter. Guidelines for resubmitting your figure files are available below the reviewer comments at the end of this letter.

If applicable, we recommend that you deposit your laboratory protocols in protocols.io to enhance the reproducibility of your results. Protocols.io assigns your protocol its own identifier (DOI) so that it can be cited independently in the future. For instructions see: https://journals.plos.org/plosone/s/submission-guidelines#loc-laboratory-protocols. Additionally, PLOS ONE offers an option for publishing peer-reviewed Lab Protocol articles, which describe protocols hosted on protocols.io. Read more information on sharing protocols at https://plos.org/protocols?utm_medium=editorial-email&utm_source=authorletters&utm_campaign=protocols.

As the corresponding author, your ORCID iD is verified in the submission system and will appear in the published article. PLOS supports the use of ORCID, and we encourage all coauthors to register for an ORCID iD and use it as well. Please encourage your coauthors to verify their ORCID iD within the submission system before final acceptance, as unverified ORCID iDs will not appear in the published article. *Only*  the individual author can complete the verification step; PLOS staff *cannot*  verify ORCID iDs on behalf of authors.

We look forward to receiving your revised manuscript.

Kind regards,

Morufu Olalekan Raimi, Ph.D

Academic Editor

PLOS One

Journal Requirements:

When submitting your revision, we need you to address these additional requirements.

1. Please ensure that your manuscript meets PLOS ONE's style requirements, including those for file naming. The PLOS ONE style templates can be found at

https://journals.plos.org/plosone/s/file?id=wjVg/PLOSOne_formatting_sample_main_body.pdf and https://journals.plos.org/plosone/s/file?id=ba62/PLOSOne_formatting_sample_title_authors_affiliations.pdf

2. Please note that PLOS One has specific guidelines on code sharing for submissions in which author-generated code underpins the findings in the manuscript. In these cases, we expect all author-generated code to be made available without restrictions upon publication of the work. Please review our guidelines at https://journals.plos.org/plosone/s/materials-and-software-sharing#loc-sharing-code and ensure that your code is shared in a way that follows best practice and facilitates reproducibility and reuse.

3. Your ethics statement should only appear in the Methods section of your manuscript. If your ethics statement is written in any section besides the Methods, please delete it from any other section.

4. In the online submission form, you indicated that de-identified qualitative data supporting the findings of this study are available from the corresponding author upon reasonable request, subject to ethical and confidentiality considerations.

All PLOS journals now require all data underlying the findings described in their manuscript to be freely available to other researchers, either 1. In a public repository, 2. Within the manuscript itself, or 3. Uploaded as supplementary information.

This policy applies to all data except where public deposition would breach compliance with the protocol approved by your research ethics board. If your data cannot be made publicly available for ethical or legal reasons (e.g., public availability would compromise patient privacy), please explain your reasons on resubmission and your exemption request will be escalated for approval.

5. If the reviewer comments include a recommendation to cite specific previously published works, please review and evaluate these publications to determine whether they are relevant and should be cited. There is no requirement to cite these works unless the editor has indicated otherwise.

Additional Editor Comments :

PLOS ONE Editorial Decision

Manuscript Number: PONE-D-26-26322

Title: Parental perceptions of children's physical activity participation: an exploration of satisfaction, school-based engagement, barriers to participation and preferred strategies for improvement.

Authors: Ojukwu CP, Okolo JC, Onyekwelu AI, Eleje C, Ekowa JL, Fatai KE

Editor: Dr. Morufu Olalekan Raimi

Decision: Major Revision

Editorial Assessment

This qualitative study explores parental perceptions of children's physical activity participation in Enugu City, Nigeria, using semi-structured interviews with 20 parents and reflexive thematic analysis guided by the Socio-Ecological Model. The research addresses an important and under-examined topic in the Sub-Saharan African context, and the five identified themes, perceptions, satisfaction, enablers, barriers, and improvement strategies, are coherently presented and well-supported by participant quotations. The manuscript is generally well-structured and clearly written. However, after careful evaluation of the revised manuscript, reviewer comments, and the authors' responses, I have identified several methodological, reporting, and conceptual issues that require attention before the manuscript can be accepted for publication. While the study has merit, the concerns raised by both reviewers, particularly regarding methodological transparency, internal inconsistencies, and the limited representativeness of the sample, are substantive and require thorough revision.

Essential Revisions

1. Resolve Internal Inconsistencies in Author Contributions and Data Collection Team

There is a clear inconsistency between the Methods section, which states that "data collection was conducted by a three-member research team comprising CO, KF, and AO," and the Authors' Contributions section, which states that "CO, OJ, and OA conducted the data collection." These are not the same individuals. Additionally, author initials for Adaeze I. Onyekwelu are used inconsistently ("OA" vs. "AO") throughout the manuscript.

Action Required: Please reconcile these discrepancies, standardise author initials, and ensure that the contributions statement accurately reflects who performed each role. This is essential for transparency and credibility.

2. Clarify the Role of Juliet Lucy Ekowa

The third-listed author does not appear to have a specific, substantive contribution beyond the generic statement that "all authors critically reviewed the manuscript." PLOS ONE's authorship criteria require each listed author to have made a substantial, specified contribution.

Action Required: Please clarify this author's specific role (e.g., data analysis, interpretation, manuscript drafting) or, if no substantive contribution was made, adjust the authorship accordingly.

3. Provide a Clearer Justification of Sample Size and Thematic Sufficiency

The authors state that recruitment continued "until thematic sufficiency was achieved" but do not explain how this was operationally judged during data collection. A post-hoc citation of sufficiency does not demonstrate methodological rigour.

Action Required: Please provide a brief account of how thematic sufficiency was determined during data collection, for example, whether coding was conducted iteratively, whether team consensus was used, and at what point it was agreed that new data were no longer yielding novel insights.

4. Address Mixed Interview Modality as a Methodological Consideration

Fourteen interviews were conducted by telephone and six face-to-face. These modalities can differ in rapport, depth of response, and ability to read non-verbal cues. The manuscript does not discuss whether modality affected data quality or how consent was documented for telephone participants specifically.

Action Required: Please add a brief statement in the Methods section describing how consent was obtained for telephone interviews (written vs. verbal) and a note in the Limitations section acknowledging the potential impact of mixed interview modalities on data richness.

5. Provide Practical Details of the Analytic Process

The Data Analysis section describes the Braun and Clarke framework at a conceptual level but lacks practical details expected by PLOS ONE reviewers. It is unclear whether coding was done manually or with software, how many transcripts were double-coded, and whether member checking was used.

Action Required: Please specify whether qualitative analysis software was used, describe the collaborative coding process (including whether independent coding was conducted), and clarify whether member checking or respondent validation was employed.

6. Strengthen Discussion of Sample Skew and Transferability

The sample was predominantly highly educated, government-sector employed, with children in private schools. The Limitations paragraph notes this, but the Discussion does not adequately address how this skew may affect the transferability of findings, particularly given that academic prioritization, a central barrier theme, may manifest differently in public schools or lower-SES families.

Action Required: Please explicitly caveat in the Discussion that the findings may not transfer to lower-SES, rural, or public-school populations. Consider moving some of this discussion from the Limitations into the main Discussion to frame interpretation more appropriately.

7. Address Minor Reporting Issues

The following minor issues should be corrected:

• Duplicate sentence: "Regular reflexive discussions and peer debriefing sessions were conducted throughout the analytic process" appears twice (Researcher Positionality and Data Analysis sections). Please remove the duplication.

• Unused abbreviations: The abbreviations list includes PE, PHE, PTA, SES, and UNEC, none of which appear to be used in the manuscript. Please remove unused abbreviations.

• Theme-closing repetitions: The summary sentences at the end of Themes 1, 3, and 4 are repetitive. Consider varying the phrasing to improve readability.

• COREQ checklist: Confirm that a completed COREQ checklist has been submitted as a supporting information file, as referenced in the Methods.

Recommendation

I recommend Major Revision. The manuscript addresses an important topic with appropriate qualitative methodology, and the findings are plausible and clearly presented. However, the internal inconsistencies, gaps in methodological detail, and insufficient handling of sample limitations require thorough attention. The authors should address the issues outlined above and respond systematically to each point in their revision.

Dr. Morufu Olalekan Raimi

Academic Editor, PLOS ONE

[Note: HTML markup is below. Please do not edit.]

Reviewers' comments:

Reviewer's Responses to Questions

**Comments to the Author**

1. Is the manuscript technically sound, and do the data support the conclusions?

Reviewer #1: Yes

Reviewer #2: Yes

**********

2. Has the statistical analysis been performed appropriately and rigorously?

Reviewer #1: Yes

Reviewer #2: Yes

**********

3. Have the authors made all data underlying the findings in their manuscript fully available?

The PLOS Data policy

Reviewer #1: Yes

Reviewer #2: Yes

**********

4. Is the manuscript presented in an intelligible fashion and written in standard English?

Reviewer #1: Yes

Reviewer #2: Yes

**********

Reviewer #1: The paper is clear and well-structured, with an important topic about parental perceptions of children’s physical activity.

Small inconsistency in author initials for data collection – please check and correct.

Timeline of interviews (2025 vs. submission in 2026) needs a short clarification.

The sample is mainly educated parents with children in private schools – it would help to note that strategies may need adaptation for public schools.

Adding one sentence on how clinicians (e.g., pediatric physiotherapists) can use these insights in practice would strengthen the conclusion.

Reviewer #2: This manuscript reports a qualitative descriptive study exploring parental perceptions of children's physical activity (PA) participation in Enugu City, Nigeria. Using semi-structured, in-depth interviews with 20 parents of nursery-, primary-, and secondary-school children, and reflexive thematic analysis guided by the Socio-Ecological Model, the authors identify five themes: context-dependent perceptions of PA participation, a continuum of parental satisfaction, perceived enablers, perceived barriers, and preferred strategies for improvement. The topic is timely and relevant given the limited qualitative evidence on parental perspectives on child PA in low- and middle-income settings, and the manuscript is generally well organised and clearly written. I have a number of methodological and reporting concerns, detailed below, that should be addressed before the manuscript is suitable for publication.

General Comments

• The study addresses an under-researched question and applies an appropriate qualitative design and an explicit theoretical framework (the Socio-Ecological Model). The five themes are coherent and well illustrated with participant quotations.

• The manuscript would benefit from greater methodological transparency in several areas (sampling rationale, analytic software, consent procedures for telephone interviews) and from resolving several internal inconsistencies in the reporting of author roles and the composition of the data-collection team (see Major Comments).

• The Discussion is comprehensive but at times restates the Results rather than critically engaging with how the findings extend, qualify, or contradict prior literature.

Major Comments

1. Inconsistent reporting of the data-collection team

The Methods state that “data collection was conducted by a three-member research team comprising CO, KF, and AO” (Data Collection Procedures), whereas the Authors’ Contributions section states that “CO, OJ, and OA conducted the data collection.” These are not the same three initials (KF vs. OJ). Please reconcile which authors actually collected the data, and correct the relevant section(s) accordingly, as this affects the credibility of the reflexivity statement built around this team.

2. Inconsistent author initials for one co-author

Adaeze I. Onyekwelu is referred to as “OA” in the design-contribution sentence and as “AO” elsewhere in the Authors’ Contributions section (and in Major Comment 1 above, in the Data Collection Procedures paragraph). Please standardise the initials used for this author throughout the contributions statement.

3. Incomplete contribution statement for one listed author

Juliet Lucy Ekowa (listed third author on the title page / fifth in the byline order) does not appear to be credited with any specific contribution (conception, design, data collection, analysis, or drafting) beyond the generic statement that “all authors critically reviewed the manuscript.” ICMJE/PLOS ONE authorship criteria require each listed author to have made a substantial, specified contribution. Please clarify this author’s role or amend the contributions statement.

4. Justification of sample size and “thematic sufficiency”

The authors state that recruitment continued “until thematic sufficiency was achieved” but do not report how this was operationally judged (e.g., were the last several transcripts coded and compared in real time, was this a team consensus, at what point in data collection was sufficiency assessed). A brief account of how sufficiency/information power was determined during data collection, rather than only cited post hoc, would strengthen the methodological rigour claim.

5. Mixed interview modality not addressed as a methodological consideration

Fourteen of twenty interviews were conducted by telephone and six face-to-face. Telephone and in-person interviews can differ in rapport, richness of response, and the interviewer’s ability to read non-verbal cues, which can affect data depth. The manuscript does not discuss whether modality affected data quality or was considered in the analysis, nor how consent was documented for telephone participants specifically (verbal vs. written). Please add a brief statement addressing this, ideally also as a limitation.

6. Analytic procedure needs more detail

The Data Analysis section describes the six-phase Braun and Clarke framework at a conceptual level but omits practical details PLOS ONE reviewers typically expect: whether coding was done manually or with qualitative analysis software (e.g., NVivo, ATLAS.ti), how many transcripts were double-coded (if any) during the reflexive discussions, and whether/how member checking or respondent validation was used. Please add these details or explain why they were not applicable to a reflexive TA approach.

7. Sample skew and generalisability

The Limitations paragraph appropriately notes that the sample was predominantly highly educated, government-sector employed, with children in private schools. Given that academic prioritisation over PA is a central barrier theme, and that private-school/higher-SES families may experience this tension differently from public-school or lower-SES families, I recommend the Discussion explicitly caveat that the barrier and satisfaction findings may not transfer to lower-SES or rural Nigerian populations, rather than deferring this entirely to the Limitations paragraph.

Minor Comments

• Title/Short Title: the full title is lengthy (24 words); consider whether it can be shortened for readability, though this is a style preference rather than a requirement.

• Abstract: consider specifying the recruitment settings (community venues) briefly in the Methods sentence of the abstract, since this is relevant to sample composition and is described in the main text.

• Table 1 and Table 2: percentages are internally consistent with the reported frequencies (verified against N = 20 for each variable); no numerical errors were identified.

• Results, Theme 1: the phrase “children’s physical activity participation is shaped by interacting influences” closely echoes similar summary sentences used at the end of Themes 3 and 4; consider varying the phrasing to avoid repetitive theme-closing statements.

• Discussion: several paragraphs (e.g., under “The Role of Family and Parental Influence”) largely restate results already presented; consider tightening these to focus on interpretation and comparison with cited literature [10–12] rather than re-describing the findings.

• Abbreviations list includes PE, PHE, PTA, SES, and UNEC, none of which appear to be used in the body of the manuscript as provided. Please remove unused abbreviations or confirm they are used in the full manuscript.

• Ethics approval number (NHREC/05/01/2008B–FWA00002458–IRB00002323) is reported identically across the submission form, Methods, and Declarations sections — consistent and appropriate.

• Please confirm that a completed COREQ checklist has been submitted as a supporting information file, as referenced in the Methods (“Reporting was guided by...COREQ”).

• Minor language check: “Regular reflexive discussions and peer debriefing sessions were conducted throughout the analytic process” appears twice in near-identical form (Researcher Positionality and Data Analysis sections); consider consolidating.

Recommendation

The manuscript addresses a relevant, under-studied question with an appropriate qualitative design, and the findings are plausible and clearly presented. However, the internal inconsistencies in author contributions/data-collection team composition, and the gaps in methodological detail (sampling sufficiency, analytic software, telephone-interview consent and modality effects), should be resolved before the manuscript can be considered further. I recommend Major Revision.

**********

what does this mean?). If published, this will include your full peer review and any attached files.

If you choose “no”, your identity will remain anonymous but your review may still be made public.

**Do you want your identity to be public for this peer review?**  For information about this choice, including consent withdrawal, please see our Privacy Policy

Reviewer #1: No

Reviewer #2: **Yes:**  Dr. Murshidul Arefin

**********

[NOTE: If reviewer comments were submitted as an attachment file, they will be attached to this email and accessible via the submission site. Please log into your account, locate the manuscript record, and check for the action link "View Attachments". If this link does not appear, there are no attachment files.]

To ensure your figures meet our technical requirements, please review our figure guidelines: https://journals.plos.org/plosone/s/figures

You may also use PLOS’s free figure tool, NAAS, to help you prepare publication quality figures: https://journals.plos.org/plosone/s/figures#loc-tools-for-figure-preparation.

NAAS will assess whether your figures meet our technical requirements by comparing each figure against our figure specifications.

# Author comment: Author response to Decision Letter 1

**DOI:** 10.1371/journal.pone.0351523.r002

27 Jul 2026

Response to Reviewer 1

We sincerely thank the reviewer for the positive evaluation of our manuscript and for the constructive comments and suggestions. We appreciate the recognition of the importance of our study and have carefully considered each recommendation. The manuscript has been revised accordingly, as detailed below.

Comment 1

The paper is clear and well-structured, with an important topic about parental perceptions of children's physical activity.

Response

We sincerely thank the reviewer for the positive assessment of our work and for recognising the importance and relevance of the study. We appreciate the encouraging comments and have carefully addressed all subsequent recommendations to further strengthen the manuscript.

Changes made

• We carefully revised the manuscript in response to all reviewer and editor comments to improve methodological transparency, reporting quality, and overall clarity.

Comment 2

Small inconsistency in author initials for data collection – please check and correct.

Response

Thank you for identifying this inconsistency. We carefully reviewed the manuscript and standardised the author initials throughout. We also reconciled the descriptions of the data collection team and revised the Authors' Contributions section to ensure consistency across all sections of the manuscript.

Changes made

• Standardised author initials throughout the manuscript.

• Revised the Data Collection Procedures section.

• Revised the Authors' Contributions section.

Comment 3

Timeline of interviews (2025 vs. submission in 2026) needs a short clarification.

Response

We appreciate this helpful suggestion. The manuscript has been revised to clarify the study timeline. We now explicitly state that interviews were conducted between August and October 2025, followed by transcription, data analysis, and interpretation between November 2025 and early 2026 prior to manuscript preparation and submission.

Changes made

• Revised the Data Collection Procedures section to clarify the study timeline.

Comment 4

The sample is mainly educated parents with children in private schools – it would help to note that strategies may need adaptation for public schools.

Response

Thank you for this valuable observation. We agree that the socioeconomic characteristics of the study sample may influence the applicability of the findings to other settings. Accordingly, we have strengthened the Discussion by explicitly acknowledging that the identified barriers, facilitators, and recommended strategies may differ among families from lower socioeconomic backgrounds, rural communities, and those with children attending public schools. We have also incorporated this consideration into the Strengths and Limitations section to provide a more balanced interpretation of the findings.

Changes made

• Revised the Discussion section.

• Revised the Strengths and Limitations section.

Comment 5

Adding one sentence on how clinicians (e.g., pediatric physiotherapists) can use these insights in practice would strengthen the conclusion.

Response

We appreciate this excellent suggestion. In response, we have expanded the "Implications for Practice and Policy" section to include the clinical relevance of the findings for paediatric physiotherapists and other child health professionals. We have also revised the Conclusion to highlight how these findings can inform the design and implementation of family-, school-, and community-based interventions aimed at promoting children's physical activity.

Changes made

• Added clinical implications to the "Implications for Practice and Policy" section.

• Revised the Conclusion to highlight the implications for paediatric physiotherapists, educators, public health practitioners, and other child health professionals.

Response to Reviewer 2

We sincerely thank the reviewer for the thorough and constructive evaluation of our manuscript. We appreciate the positive comments regarding the importance of the research topic, the appropriateness of the qualitative design, and the coherence of the findings. We have carefully considered each comment and have revised the manuscript accordingly. Our detailed responses are provided below.

General Comment

The study addresses an under-researched question and applies an appropriate qualitative design and an explicit theoretical framework (the Socio-Ecological Model). The five themes are coherent and well illustrated with participant quotations.

Response

We sincerely thank the reviewer for these encouraging comments and for recognising the importance of the study, the suitability of the qualitative design, and the application of the Socio-Ecological Model. We appreciate the constructive recommendations provided and have revised the manuscript to improve methodological transparency, reporting quality, and interpretation of the findings.

Major Comments

Comment 1

Inconsistent reporting of the data-collection team.

Response

We thank the reviewer for identifying this important inconsistency. The manuscript has been carefully reviewed and revised to ensure that the composition of the data collection team is reported consistently throughout. The Data Collection Procedures, Researcher Positionality, and Authors' Contributions sections have all been updated to accurately reflect the roles of the researchers involved in the qualitative study.

Changes made

• Revised the Data Collection Procedures.

• Revised the Researcher Positionality section.

• Revised the Authors' Contributions section.

• Standardised author initials throughout the manuscript.

Comment 2

Inconsistent author initials for one co-author.

Response

Thank you for bringing this to our attention. We have standardised the initials of Adaeze I. Onyekwelu throughout the manuscript to ensure consistency across all sections, including the Authors' Contributions statement.

Changes made

• Standardised author initials throughout the manuscript.

Comment 3

Incomplete contribution statement for one listed author.

Response

We appreciate this important observation. The Authors' Contributions section has been revised to clearly specify Juliet Lucy Ekowa's contributions to the study. Her role now includes contributing to the development and refinement of the research questions and study design, as well as participation in data analysis and interpretation, thereby ensuring compliance with the PLOS ONE authorship criteria.

Changes made

• Revised the Authors' Contributions section.

Comment 4

Justification of sample size and thematic sufficiency.

Response

We thank the reviewer for this valuable recommendation. The Participant Selection and Sampling Strategy section has been expanded to provide a clearer description of how thematic sufficiency was determined during data collection. Specifically, we now explain that interview transcripts were reviewed alongside ongoing interviews, preliminary coding and discussion of emerging concepts were undertaken iteratively by the research team, and recruitment ceased when successive interviews yielded no substantially new themes, with later interviews contributing additional depth and clarification rather than novel conceptual insights.

Changes made

• Expanded the Participant Selection and Sampling Strategy section.

Comment 5

Mixed interview modality not addressed as a methodological consideration.

Response

We appreciate this helpful suggestion. The Methods section has been revised to clarify the procedures used for telephone interviews, including the electronic distribution and return of participant information sheets and signed consent forms before interviews commenced. We have also expanded the Strengths and Limitations section to acknowledge that the use of both telephone and face-to-face interviews may have influenced data richness because telephone interviews limited the observation of non-verbal cues, while also improving accessibility and participation among parents with demanding schedules.

Changes made

• Revised the Data Collection Procedures.

• Revised the Strengths and Limitations section.

Comment 6

Analytic procedure needs more detail.

Response

We thank the reviewer for this constructive recommendation. The Data Analysis section has been substantially expanded to provide greater methodological transparency. We now specify that coding was undertaken manually, describe the iterative coding process, explain the collaborative reflexive discussions undertaken by the research team during code and theme development, and clarify that the analysis followed Braun and Clarke's Reflexive Thematic Analysis. We also clarify that qualitative data analysis software was not used and that participant (member) checking was not undertaken, consistent with the principles of reflexive thematic analysis.

Changes made

• Revised the Data Analysis section.

Comment 7

Sample skew and generalisability.

Response

We appreciate this insightful recommendation. The Discussion has been strengthened by explicitly acknowledging that the participant sample consisted predominantly of highly educated parents employed in the government sector, many of whom had children attending private schools. We now discuss how these characteristics may influence parental perceptions of children's physical activity and recognise that the findings may not be directly transferable to lower socioeconomic populations, rural communities, or families with children attending public schools. Similar clarification has also been incorporated into the Strengths and Limitations section.

Changes made

• Revised the Discussion.

• Revised the Strengths and Limitations section.

Minor Comments

Comment 1

The title is rather long.

Response

We appreciate the reviewer's suggestion. We carefully considered shortening the title but elected to retain it because we believe it accurately reflects the scope, objectives, and principal findings of the study while remaining informative for readers.

Comment 2

Specify the recruitment settings in the abstract.

Response

Thank you for this helpful suggestion. The Abstract has been revised to specify that participants were recruited purposively from community settings, including churches, marketplaces, shopping centres, and recreational areas within Enugu City.

Changes made

• Revised the Abstract.

Comment 3

Theme-closing summaries are repetitive.

Response

We appreciate this observation. The concluding summary statements for Themes 1, 3, and 4 have been revised to improve readability, reduce repetition, and strengthen the interpretive nature of the findings.

Changes made

• Revised the Results section.

Comment 4

Discussion restates the Results.

Response

Thank you for this constructive recommendation. The Discussion has been revised to reduce repetition of the Results section and place greater emphasis on interpretation, integration with previous literature, and implications for practice and policy.

Changes made

• Revised the Discussion.

Comment 5

Unused abbreviations.

Response

Thank you for identifying this issue. We reviewed the abbreviations list and removed abbreviations that were not used in the manuscript to improve clarity and consistency.

Changes made

• Revised the abbreviations list.

Comment 6

COREQ checklist.

Response

Thank you for this reminder. A completed and updated COREQ checklist has been prepared and included as Supporting Information. The checklist has been reviewed to ensure consistency with the revised manuscript.

Changes made

• Submitted the completed COREQ checklist as Supporting Information.

Comment 7

Duplicate reflexivity statement.

Response

We appreciate the reviewer's careful reading of the manuscript. The duplicated statement regarding reflexive discussions and peer debriefing has been removed, and the relevant sections have been revised to improve clarity while avoiding unnecessary repetition.

Changes made

• Revised the Researcher Positionality and Data Analysis sections.

General Response

We sincerely thank the Academic Editor and both reviewers for their thoughtful and constructive comments. Their suggestions have substantially improved the methodological transparency, reporting quality, and overall clarity of our manuscript. We believe that the revised manuscript addresses all concerns raised and hope that it is now suitable for publication in PLOS ONE.

Attachment Submitted filename: RESPONSE TO THE ACADEMIC EDITOR AND REVIEWERS.docx

# Editor report: Decision Letter 1

**DOI:** 10.1371/journal.pone.0351523.r003

**Authors:** Morufu Raimi

3 Aug 2026

Dear Dr. Fatai,

Thank you for submitting your manuscript to PLOS ONE. After careful consideration, we feel that it has merit but does not fully meet PLOS ONE’s publication criteria as it currently stands. Therefore, we invite you to submit a revised version of the manuscript that addresses the points raised during the review process.

Please submit your revised manuscript by Sep 02 2026 11:59PM. If you will need more time than this to complete your revisions, please reply to this message or contact the journal office at plosone@plos.org. When you're ready to submit your revision, log on to https://www.editorialmanager.com/pone/ and select the 'Submissions Needing Revision' folder to locate your manuscript file.

- A letter that responds to each point raised by the academic editor and reviewer(s). You should upload this letter as a separate file labeled 'Response to Reviewers'.
- A marked-up copy of your manuscript that highlights changes made to the original version. You should upload this as a separate file labeled 'Revised Manuscript with Track Changes'.
- An unmarked version of your revised paper without tracked changes. You should upload this as a separate file labeled 'Manuscript'.

If you would like to make changes to your financial disclosure, please include your updated statement in your cover letter. Guidelines for resubmitting your figure files are available below the reviewer comments at the end of this letter.

If applicable, we recommend that you deposit your laboratory protocols in protocols.io to enhance the reproducibility of your results. Protocols.io assigns your protocol its own identifier (DOI) so that it can be cited independently in the future. For instructions see: https://journals.plos.org/plosone/s/submission-guidelines#loc-laboratory-protocols. Additionally, PLOS ONE offers an option for publishing peer-reviewed Lab Protocol articles, which describe protocols hosted on protocols.io. Read more information on sharing protocols at https://plos.org/protocols?utm_medium=editorial-email&utm_source=authorletters&utm_campaign=protocols.

As the corresponding author, your ORCID iD is verified in the submission system and will appear in the published article. PLOS supports the use of ORCID, and we encourage all coauthors to register for an ORCID iD and use it as well. Please encourage your coauthors to verify their ORCID iD within the submission system before final acceptance, as unverified ORCID iDs will not appear in the published article. *Only*  the individual author can complete the verification step; PLOS staff *cannot*  verify ORCID iDs on behalf of authors.

We look forward to receiving your revised manuscript.

Kind regards,

Morufu Olalekan Raimi, Ph.D

Academic Editor

PLOS One

Journal Requirements:

If the reviewer comments include a recommendation to cite specific previously published works, please review and evaluate these publications to determine whether they are relevant and should be cited. There is no requirement to cite these works unless the editor has indicated otherwise.

Please review your reference list to ensure that it is complete and correct. If you have cited papers that have been retracted, please include the rationale for doing so in the manuscript text, or remove these references and replace them with relevant current references. Any changes to the reference list should be mentioned in the rebuttal letter that accompanies your revised manuscript. If you need to cite a retracted article, indicate the article’s retracted status in the References list and also include a citation and full reference for the retraction notice.

Additional Editor Comments (if provided):

Editorial Decision Letter

PLOS ONE – Earth & Environmental Sciences

Manuscript ID: PONE-D-26-26322_R1

Title: Parental perceptions of children's physical activity participation: an exploration of satisfaction, school-based engagement, barriers to participation and preferred strategies for improvement

Authors: Ojukwu CP, Okolo JC, Onyekwelu AI, Eleje C, Ekowa JL, Fatai KE

Decision: Minor Revisions Required

Date: August 3, 2026

To: Kadiree Ekundayo Fatai, David Umahi, Federal University of Health Sciences

From: Dr. Morufu Olalekan Raimi, PhD

Academic Editor, PLOS ONE

1. Decision Rationale

Thank you for submitting your revised manuscript and the comprehensive point-by-point response. I acknowledge the substantial effort invested in addressing the methodological and reporting concerns raised during the previous review. The manuscript has improved considerably, with clearer articulation of the analytical process, strengthened discussion of sample limitations, and resolution of most internal inconsistencies. The study addresses an important and under-researched topic, parental perceptions of children's physical activity in a Nigerian urban context, and the application of reflexive thematic analysis within the Socio-Ecological Model framework is appropriate. The five identified themes are coherent and well-supported by participant quotations.

However, despite the revisions, several issues persist that require attention before the manuscript can be accepted. These are primarily related to incomplete resolution of author contribution inconsistencies, remaining duplication in the Data Analysis section, and minor but important methodological clarifications. These issues are remediable and do not require additional data collection. I therefore request a Minor Revision to address the specific points below.

2. Mandatory Revisions

Revision A: Resolve Remaining Author Contribution Inconsistencies (Critical)

Your response letter and the revised manuscript indicate that the data collection team comprised CO, OJ, CE, and AO (p. 18, line 186-187). However, the Authors' Contributions section (p. 36, lines 540–545) still states:

“CO, OJ, CE, and AO conducted the data collection.”

Yet the Data Collection Procedures section (p. 18, line 188) now states:

“Data collection was conducted by a three-member research team comprising CO, KF, and AO.”

This remains inconsistent. KF and CE are not the same individuals. Please:

1. Reconcile this discrepancy definitively. Specify exactly which authors collected the data.

2. Ensure the Authors' Contributions section accurately reflects the roles of all authors, including Juliet Lucy Ekowa, whose specific contribution remains vaguely described as "contributing to the development and refinement of the research questions and study design, as well as participation in data analysis and interpretation." If she made these contributions, state this clearly in the section.

Required Action: Revise both sections to ensure they are consistent and accurately reflect who performed which roles.

Revision B: Remove Duplicate Text in Data Analysis Section

The Data Analysis section (p. 19, lines 220-239 and p. 54, lines 241-258) contains extensive duplication. For example, the description of the six-phase Braun and Clarke framework appears twice in near-identical form. This duplication is unnecessary and should be removed.

Required Action: Consolidate the Data Analysis section into a single, concise description of the analytical process. Remove all redundant text.

Revision C: Clarify Thematic Sufficiency Operationalisation

Your response states that "recruitment ceased when successive interviews yielded no substantially new themes." However, the revised manuscript still does not clearly explain how this was judged during data collection. Please add a brief statement in the Participant Selection and Sampling Strategy section explaining:

• Whether coding was conducted iteratively alongside data collection.

• Whether team consensus was used to determine when sufficiency was reached.

• At what point (e.g., after how many interviews) the team agreed that new data were no longer yielding novel insights.

Required Action: Add a concise statement in the Methods section describing the operationalisation of thematic sufficiency.

Revision D: Address Mixed Interview Modality More Explicitly

You have added a statement in the Limitations section acknowledging that telephone interviews may have limited observation of non-verbal cues. However, the Methods section (p. 18, lines 183–185) still does not clearly describe how consent was documented for telephone participants. You state that consent forms were sent via WhatsApp and returned electronically, but please clarify:

• Were these signed consent forms returned before or after the interview?

• Was verbal consent also obtained at the start of the telephone interview?

Required Action: Add a clear statement in the Data Collection Procedures section describing the consent process for telephone participants.

Revision E: Final Language and Consistency Edits

1. Table 1: The table is well-presented. However, the variable “Parent's Occupation” is listed in the table but not described in the text (p. 21, lines 242-255). Please either describe this variable in the text or remove it from the table if it was not used in the analysis.

2. Abbreviations List: You have removed unused abbreviations. However, “PHE” (Physical and Health Education) appears in the list but is not used in the manuscript. Please remove it.

3. Duplicate Reflexivity Statement: You have removed the duplication in the Researcher Positionality section, but the Data Analysis section still contains a near-identical sentence (p. 54, lines 256-258). Please remove this duplication.

3. Editorial Conclusion

This manuscript makes a valuable contribution to the limited qualitative evidence on parental perceptions of children's physical activity in sub-Saharan Africa. The revised manuscript is substantially improved and now meets most of the journal's standards for methodological transparency and reporting quality. The remaining issues are limited to incomplete resolution of author contribution inconsistencies, duplication in the Data Analysis section, and minor but important methodological clarifications. These can be resolved without additional data collection or substantive rewriting.

Final Decision: Minor Revisions Required

Please resubmit your revised manuscript within 14 days of this letter. Include a detailed, point-by-point response to each comment and use tracked changes to highlight all modifications in the revised manuscript.

I look forward to receiving your revised submission.

Sincerely,

Dr. Morufu Olalekan Raimi, PhD

Academic Editor, PLOS ONE

# Author comment: Author response to Decision Letter 2

**DOI:** 10.1371/journal.pone.0351523.r004

5 Aug 2026

No response

Attachment Submitted filename: RESPONSE TO THE ACADEMIC EDITOR - 5-8-26.docx

# Editor report: Decision Letter 2

**DOI:** 10.1371/journal.pone.0351523.r005

**Authors:** Morufu Raimi

16 Aug 2026

Parental perceptions of children's physical activity participation: an exploration of satisfaction, school-based engagement, barriers to participation and preferred strategies for improvement.

PONE-D-26-26322R2

Dear Authors,

We’re pleased to inform you that your manuscript has been judged scientifically suitable for publication and will be formally accepted for publication once it meets all outstanding technical requirements.

Within one week, you’ll receive an e-mail detailing the required amendments. When these have been addressed, you’ll receive a formal acceptance letter and your manuscript will be scheduled for publication.

An invoice will be generated when your article is formally accepted. Please note, if your institution has a publishing partnership with PLOS and your article meets the relevant criteria, all or part of your publication costs will be covered. Please make sure your user information is up-to-date by logging into Editorial Manager at Editorial Manager® and clicking the ‘Update My Information' link at the top of the page. For questions related to billing, please contact billing support.

If your institution or institutions have a press office, please notify them about your upcoming paper to help maximize its impact. If they’ll be preparing press materials, please inform our press team as soon as possible -- no later than 48 hours after receiving the formal acceptance. Your manuscript will remain under strict press embargo until 2 pm Eastern Time on the date of publication. For more information, please contact onepress@plos.org.

Kind regards,

Morufu Olalekan Raimi, Ph.D

Academic Editor

PLOS One

Additional Editor Comments (optional):

EDITOR'S DECISION

Manuscript: Decomposing socioeconomic inequalities in COVID-19 vaccination uptake among Nigerian women of reproductive age: a further analysis of population-based data

Manuscript Number: PONE-D-26-20978R3

Journal: PLOS ONE

Academic Editor: Dr. Morufu Olalekan Raimi, PhD

Recommendation: Accept

1. EXECUTIVE SUMMARY

This manuscript examines socioeconomic inequalities in COVID-19 vaccination uptake among 36,555 Nigerian women of reproductive age using 2024 NDHS data. The study applies Erreygers concentration indices and decomposition analysis to identify drivers of inequality. The author has now completed three rounds of revision, addressing critical methodological concerns including decomposition equation specification, wife-beating variable coding, and causal language.

Strengths:

• Nationally representative dataset (36,555 women, 93.6% response rate)

• Appropriate application of Erreygers decomposition methodology

• Complete transparency: analytic dataset, code, and questionnaire provided

• Clear policy implications for vaccine equity in LMICs

• Thorough response to previous review rounds

Resolution of Prior Concerns:

• Decomposition equations now mathematically correct with AMEs explicitly annotated

• Wife-beating variable coding clarified with clear conceptual rationale

• Causal language removed throughout; associations appropriately framed

• Data availability statement clearly distinguishes analytic dataset from source data

• Abstract and terminology inconsistencies corrected

2. ASSESSMENT OF REVISIONS

2.1 Decomposition Model Specification (Revision A)

Status: Resolved

The author has corrected all decomposition equations. Key improvements:

1. AME annotation: A clear statement now precedes the equations specifying that average marginal effects (AMEs), not raw logistic coefficients, are entered as βₖ in the decomposition. This addresses the fundamental distinction between log-odds and probability-scale effects.

2. Error term notation: Consistently written as GCε throughout, replacing the previous mathematically incorrect notation.

3. Erreygers correction: The full formula is now provided with explicit statement that correction follows Erreygers (2009) for a binary outcome bounded between 0 and 1. The correction factor 4/(b-a) is correctly specified.

4. AME explanation placement: Moved immediately before Equation (1) as requested, improving logical flow.

Verification: Equations now accurately reflect:

• Linear additive specification with AMEs

• Correct concentration index decomposition

• Erreygers normalization for bounded binary outcomes

2.2 Wife-Beating Variable Coding (Revision B)

Status: Resolved

The author has provided explicit rationale in the Methods section:

"Higher acceptance of wife-beating is considered an indicator of lower women's empowerment because it reflects the adherence to gender norms that allows male dominance and violence against women."

Key clarifications:

• Variable relabelled as “Community-level acceptance of wife-beating” throughout

• Table labels now clearly distinguish “More acceptance (less empowered)" and "Less acceptance (more empowered)”

• Results and Discussion explicitly interpret the coding direction

• 79% cut-point justified with full distribution statistics (median, IQR, min, max) provided

Verification: The coding is now transparent and conceptually defensible. The rationale that acceptance of wife-beating reflects internalized patriarchal norms and reduced autonomy is well-supported by the literature cited (Singh et al., 2013; Alabi & Ramsden, 2021; WHO, 2021).

2.3 Causal Language (Revision C)

Status: Resolved

All identified causal statements have been revised:

Original Revised

"could enhance both access to and acceptance" "may be associated with access to and acceptance"

"socioeconomic status may be associated with the need for a critical role in shaping access" "socioeconomic status may be associated with access to and utilization of COVID-19 vaccination services"

"where the well-off are better positioned" "where the well-off may be better positioned"

Verification: The manuscript consistently uses associative language appropriate for cross-sectional design. No remaining causal claims.

2.4 Data Availability Statement (Revision D)

Status: Resolved

The statement now clearly distinguishes:

• Analytic dataset: https://doi.org/10.6084/m9.figshare.32936522

• Original DHS data: https://www.dhsprogram.com/data/dataset/Nigeria_Standard-DHS_2024.cfm?flag=1

Verification: Complete transparency achieved.

2.5 Language and Consistency (Revision E)

Status: Resolved

• Abstract revised to: "Approximately one-third of the least disadvantaged, one-quarter of the moderately disadvantaged, and one-fifth of the most disadvantaged women reported receiving a COVID-19 vaccine."

• "Socioeconomically" (adverb) and “socioeconomic” (adjective) now used consistently throughout.

3. REMAINING OBSERVATIONS (Not Requiring Revision)

3.1 Duplicate Text in Abstract

The abstract contains a minor duplication: the sentence about one-third/one-quarter/one-fifth appears twice (Lines 58-63). This appears to be a formatting error from the marked-up copy and can be corrected during typesetting. It does not affect the scientific content.

3.2 Duplicate References

Reference 24 appears twice (Page 35). This is a minor reference list duplication that can be corrected during production.

3.3 Duplicate Text in Methods

There is a minor repetition in the Analytical approach section (duplicate paragraphs on concentration index and AMEs). This appears to be a copy-paste artifact from the revision process. Production editing will resolve this.

4. OVERALL ASSESSMENT

4.1 Scientific Contribution

This manuscript makes a valuable contribution to the literature on vaccine equity in Nigeria. The application of Erreygers decomposition to the 2024 NDHS data provides rigorous quantification of inequality drivers. Key findings that higher education, media exposure, employment, health insurance, and southern geopolitical zones contribute positively to inequality offer actionable insights for targeted interventions.

4.2 Methodological Rigor

The methodology is sound. The author has:

• Used appropriate survey weighting (svy function)

• Employed Erreygers concentration indices for binary outcomes

• Used AMEs in decomposition (correct approach)

• Provided complete code and data for reproducibility

• Addressed all reviewer concerns substantively

4.3 Response to Reviewers

The author has responded comprehensively to all three rounds of review. The current revision demonstrates:

• Careful attention to mathematical correctness

• Transparency about coding decisions

• Appropriate framing of associational findings

• Complete data and code availability

5. DECISION

Recommendation: Accept

The manuscript is scientifically sound, methodologically rigorous, and makes a meaningful contribution to understanding vaccine equity in Nigeria. All reviewer concerns have been addressed substantively. The remaining minor formatting issues (duplicate text in abstract, duplicate reference) can be corrected during production and do not warrant additional revision.

Editor Signature: Dr. Morufu Olalekan Raimi, PhD

Date: August 13, 2026

Action: Accept

Confidential Comments to Production Team: Please note the following minor corrections during typesetting:

1. Abstract duplication (Lines 58-63): Remove duplicate sentence

2. Reference 24 duplication: Remove duplicate entry

3. Duplicate paragraphs in Analytical approach section: Consolidate

6. FINAL NOTE TO AUTHOR

Congratulations on completing a rigorous revision process. This manuscript will make a valuable contribution to the literature on vaccine equity in Nigeria and has clear policy relevance. I appreciate your thorough engagement with the review process and your commitment to methodological transparency. I look forward to seeing this work in print.

Reviewers' comments:

# Editor report: Acceptance letter

**DOI:** 10.1371/journal.pone.0351523.r006

**Authors:** Morufu Raimi

PONE-D-26-26322R2

PLOS One

Dear Dr. Fatai,

I'm pleased to inform you that your manuscript has been deemed suitable for publication in PLOS One. Congratulations! Your manuscript is now being handed over to our production team.

At this stage, our production department will prepare your paper for publication. This includes ensuring the following:

* All references, tables, and figures are properly cited

* All relevant supporting information is included in the manuscript submission,

* There are no issues that prevent the paper from being properly typeset

You will receive further instructions from the production team, including instructions on how to review your proof when it is ready. Please keep in mind that we are working through a large volume of accepted articles, so please give us a few days to review your paper and let you know the next and final steps.

Lastly, if your institution or institutions have a press office, please let them know about your upcoming paper now to help maximize its impact. If they'll be preparing press materials, please inform our press team within the next 48 hours. Your manuscript will remain under strict press embargo until 2 pm Eastern Time on the date of publication. For more information, please contact onepress@plos.org.

You will receive an invoice from PLOS for your publication fee after your manuscript has reached the completed accept phase. If you receive an email requesting payment before acceptance or for any other service, this may be a phishing scheme. Learn how to identify phishing emails and protect your accounts at https://explore.plos.org/phishing.

If we can help with anything else, please email us at customercare@plos.org.

Thank you for submitting your work to PLOS One and supporting open access.

Kind regards,

PLOS ONE Editorial Office Staff

on behalf of

Prof Morufu Olalekan Raimi

Academic Editor

PLOS One
